# Human Health Impacts of Residential Radon Exposure: Updated Systematic Review and Meta-Analysis of Case–Control Studies

**DOI:** 10.3390/ijerph20010097

**Published:** 2022-12-21

**Authors:** Le Thi Nhu Ngoc, Duckshin Park, Young-Chul Lee

**Affiliations:** 1 Department of Industrial and Environmental Engineering, Graduate School of Environment, Gachon University, 1342 Seongnam-daero, Seongnam-si 13120, Republic of Korea; 2 Korea Railroad Research Institute (KRRI), 176 Cheoldobakmulkwan-ro, Uiwang-si 16105, Republic of Korea; 3 Department of BioNano Technology, Gachon University, 1342 Seongnam-daero, Seongnam-si 13120, Republic of Korea

**Keywords:** radon risk, lung cancer, childhood leukemia, case–control studies

## Abstract

This study investigated the impact of residential radon exposure on human cancers (i.e., lung cancer and childhood leukemia) through a systematic review and meta-analysis of case–control studies. A total of 9724 articles obtained from electronic databases were assessed; however, only 55 case–control studies were eligible after manually screening and eliminating unnecessary studies. The causal associations were addressed by determining the meta-analysis’s estimated size effects (i.e., ORs/RRs) of the meta-analysis. Residential radon was revealed to significantly increase the incidence of lung cancer and childhood leukemia with pooled ORs of 1.38 [1.19; 1.60] (I^2^ = 90%; *p* < 0.00001) and 1.43 [1.19; 1.72] (I^2^ = 0% and *p* = 0.51), respectively. In addition, subgroup analyses were performed to reduce the heterogeneity of the initial meta-analyses. The results provided strong evidence that inhaling radon in the indoor environments is closely associated with the development of lung cancer and childhood leukemia in patients living in Europe and areas with high radon levels (≥100 Bq/m^3^).

## 1. Introduction

Radon is well known as a naturally occurring radioactive gas that may be found in high concentrations in indoor environments such as homes, schools, and workplaces. The primary sources of residential radon are soils and rocks around the foundation, fuels, building materials, and domestic water [1]. The indoor radon concentration can vary greatly from 10 Bq/m^3^ to more than 10,000 Bq/m^3^ (from 0.27 pCi/L to 270 pCi/L) [1]. Indoor radon escapes from the ground into the air, where it decays and produces other radioactive particles [1,2]. When we breathe, these particles are deposited on the cells lining the airways, where they can damage DNA and potentially cause cancers [1,2]. 

Lung cancer is the most commonly diagnosed cancer worldwide and the leading cause of cancer death [3]. According to Global Cancer Observatory (2020), lung cancer accounted for 11.4% (2.2 million) of total cancer cases and 18% (1.79 million) of cancer deaths [3]. Among the risk factors of lung cancer (e.g., radon, air pollution, arsenic, silica, diesel, chromium, cadmium, and beryllium), radon is well known as one of the 19 environmental carcinogens recognized by the World Health Organization (WHO). The WHO reports that radon causes up to 3–14% of all lung cancers in a country, depending on the country’s average radon level and smoking prevalence [1]. Moreover, radon accounts for 2% (21,000 deaths) of European cancer [3]. The risk of lung cancer increases by approximately 16% per 100 Bq/m^3^ increase in long-time average radon concentration [1]. Studies on residential radon as a risk factor for lung cancer have attracted attention and provided substantial epidemiological evidence. For instance, Darby et al. (1998) conducted a meta-analysis analysis from 13 studies in Europe and demonstrated a statistical increase of 16% in lung cancer risk per 100 Bq/m^3^ of residential radon [4]. Lorenzo-Gonzalez et al. (2019) found an increased odds ratio (OR) for lung cancer in 523 individuals exposed to radon ≥200 Bq/m^3^ compared with those exposed to ≤100 Bq/m^3^ [5]. It has been reported that the role of radon in the development of lung cancer is related to the emission of alpha particles from radon with a high potential for damage to the respiratory epithelium [6]. First, alpha particles can impact the respiratory epithelium and produce various cytotoxic and genotoxic effects, which favor carcinogenesis [6]. These genotoxic effects result in large-scale molecular changes, including DNA double-strand breaks, translocations and deletions, substitutions, and chromosomal rearrangements, thereby inducing the dysregulation of cytokines and the increased production of proteins associated with carcinogenesis [6]. Secondly, alpha radiation impacts the immune system in the tumor microenvironment [6]. The overproduction of reactive oxygen species (ROS) in the lungs caused by repeated exposure to radon can cause oxidative stress, leading to pulmonary inflammation. Moreover, radon can enhance tumor immunogenicity by increasing genomic instability and cluster mutations in the tumor cells [6].

Childhood leukemias are a hematological cancer of the leukocytes in children [7]. Diseases that lead to the dramatically excessive proliferation of white blood cells, resulting in an invasion of the bone marrow, the lymph nodes, and the spleen with an excessive accumulation of cells in the blood flow, end with an extension of malignant colonization in various tissues of the body, especially the liver and the central nervous system [7]. Childhood leukemia is commonly induced by genetic risk factors (e.g., genetic syndromes and inherited immune system problems) and environmental factors (ionizing/non-ionizing radiation exposure, chemotherapy exposure, prenatal environment, socioeconomic status, and immune system suppression) [7,8]. Regarding environmental risk factors, especially radon exposure, the exact mechanisms underlying leukemia remain unclear. It is hypothesized that when we breathe, a small amount of radon is transported to the red bone marrow, which then causes specific changes in the DNA, especially the creation of double-strand DNA breaks inside normal bone marrow cells. Once the leukocytes in blood cells undergo an out-of-control mutation, they become leukemia cells. Recently, it has been reported that natural background radon contributes to a 20% increase in childhood leukemia cases [9,10]. A causal relationship has been established between residential radon and childhood leukemia, with side-effects depending on the age at the time of exposure, sex, and exposure duration through many studies [9,11]. For example, Axelson et al. (2002) evaluated the possible risk of lymphocytic leukemia from exposure to residential radon in 312 cases and 1418 controls in Sweden during 1980–1989. The study obtained a conditional OR of 1.4 [1.05; 1.91], which suggested some risk of acute lymphocytic leukemia in children from indoor ionizing radiation [12]. Brauner et al. (2010) reported a risk for childhood leukemia associated with a radon increase of 10^3^ Bq/m^3^ per year with an OR of 1.77 [1.11; 2.83], proposing a high risk of radon exposure causing childhood leukemia [12].

In this light, numerous recent case–control studies have addressed the hypothesis that adults and children who grow up in homes with elevated radon concentrations have an increased risk of developing lung cancer and childhood leukemia, respectively [11,13,14,15,16]. However, these studies reported different conclusions with different effect sizes. Furthermore, these studies had a heterogeneous study population, design, setting, exposure measurement, and risk calculation method, making it difficult to draw consistent conclusions. Therefore, systematic reviews and meta-analyses have been applied to pool individual estimated size effects and to provide a statistically significant conclusion to the hypothesis.

Many recent systematic reviews and meta-analyses have addressed this topic; however, they include very little eligible data, and therefore, their results may be underestimated [2,16,17]. Moreover, the number of case–control studies has been increasing year by year; therefore, this updated systematic review and meta-analysis were designed to access a more significant, larger sample size and higher-quality data source to provide more accurate results than the previous systematic reviews. This study collected and analyzed estimated size effects, namely, the odds ratio (OR) and risk ratio (RR), from case–control studies of lung cancer and childhood leukemia caused by radon. The results from traditional comparisons and subgroup analyses might contribute to discovering the impact of radon exposure on human cancers.

## 2. Materials and Methods

### 2.1. Literature Search Strategy

According to the Preferred Reporting Items for Systematic Reviews and Meta-analysis (PRISMA) protocol 2009 [18], a literature search on the impacts of residential radon on human health was performed in the English-language databases, including PubMed, Cochrane Library, EMBASE, MedRxiv, and Elsevier, to obtain relevant articles published up to 2022. The literature search was performed using the following keywords: residential radon, human health, lung cancer, childhood leukemia, and case–control studies. The protocol has been registered with the PROSPERO International Prospective Register of Systematic Reviews. The registration number is CRD42022379937.

### 2.2. Study Selection and Data Extraction

#### 2.2.1. Study Selection

All case–control studies that evaluated the adverse effects of residential radon exposure on human health, particularly lung cancer and childhood leukemia, were included in this study. All case–control studies that studied at least one case (lung cancer or childhood leukemia patient) and one matched participant in the control arm were considered as long as there were no differences in the relationship between residential radon exposure and human cancers. Case–control studies with multiple components were considered eligible if they directly compared the case and the control, with the right arms included in the meta-analysis. In the case–control studies, results were reported as correlation coefficients (i.e., ORs/RRs) with their corresponding 95% confidence interval (CI) regardless of whether it was the primary outcome or not. No restrictions were set on the case–control status, patient background, indoor environmental quality, and geographical region. Furthermore, the independent and dependent variables for case–control studies should be measured for the individual radon doses and the incidence of lung cancer and childhood leukemia, respectively. If multiple studies reported different outcome types (e.g., incidence or mortality), the study with incidence outcomes was selected.

Studies of workplace exposures (e.g., studies of miners) and studies of different types (e.g., ecological, cohort, and randomized clinical trials) were excluded. Duplicate articles from all or part of other articles were excluded. Among repeated articles, only the latest one was included. Studies that only investigated the exposure to gamma radiation and radon daughters were excluded. The hypothesis of this study was to confirm a causal relationship between residential radon exposure and the incidence of human cancers. In addition, studies of the relationship between exposure to radon in water and cancers were excluded. It can explain that radon needs to be in gaseous form to have remarkably carcinogenic effects on the lungs. In contrast, radon in drinking water is associated with an increased risk of developing other organ cancers, primarily stomach cancer [12]. In terms of water containing radon being used in the home for cooking, washing dishes, and showering, radon gas escapes from the water, goes into the air, and then traps in the lungs. However, it is reported that a minimal amount of radon is transferred from domestic water to indoor air, with a 37 Bq/m^3^ increase in radon in indoor air from a 370,000 Bq/m^3^ increase in dissolved radon in domestic water [12]. Therefore, radon escaping from water is not a massive risk of developing lung cancer. Thus, a separate study should be performed for the risk of water-soluble radon because the dose–response relationship would be significantly different from the threat of radon in indoor air.

#### 2.2.2. Data Extraction

Two independent reviewers screened each included study and recorded appropriate data for this systematic review and meta-analysis. Then, a predefined form was used to extract the following data from each case–control study: author name, year of publication, country of study, period of investigation, the age of participant, sample size (cases and controls), types of cancer (i.e., lung cancer and childhood leukemia), residential radon levels (Bq/m^3^), estimated effect sizes (i.e., OR and RR), and adjustment criteria for the main confounding factor of interest.

### 2.3. Meta-Analysis

For all dichotomous outcomes, the estimated size effects (i.e., ORs/RRs) on the lung cancer/childhood leukemia incidence in participants with the highest radon exposure relative to the participants with no/lowest radon exposure were pooled using the random-effects model and expressed as the OR/RR with 95% CI [19]. If the CI for an estimate includes 1, it cannot indicate a statistically significant difference between the groups being compared; in contrast, if it does not include 1, it indicates a statistically significant difference [19]. Initially, all studies were pooled together, and a sensitivity analysis was performed to assess the influences of the methodological concerns by grouping them into subgroups, including geographical region, period of investigation, residential radon level, and smoking status. In addition, the heterogeneity of each study was analyzed using the standard coefficient heterogeneity (I^2^) test. Heterogeneity was considered negligible, moderate, and large when I^2^ was less than 25%, 25–50%, and more than 50%, respectively [19]. The value *p* ≥ 0.10 was considered statistically significant, and it suggested that the studies were homogeneous. All these analyses were conducted using Comprehensive Meta-Analysis Review Manager (version 5.3, Copenhagen, Denmark: The Nordic Cochrane Center, The Cochrane Collaboration, 2014 version V3, Biostat Inc., Tampa, FL, USA).

### 2.4. Quality Assessment of Included Studies

To explore the validity of eligible case–control studies, the quality of biased evaluations was determined according to the Newcastle–Ottawa scale guideline [20]. A “star system” was developed, in which a study was judged on three broad perspectives: the selection of study groups, comparability of study groups, and ascertainment of radon exposure (Table S1).

## 3. Results and Discussion

### 3.1. Characteristics of Included Studies

**Figure 1:**
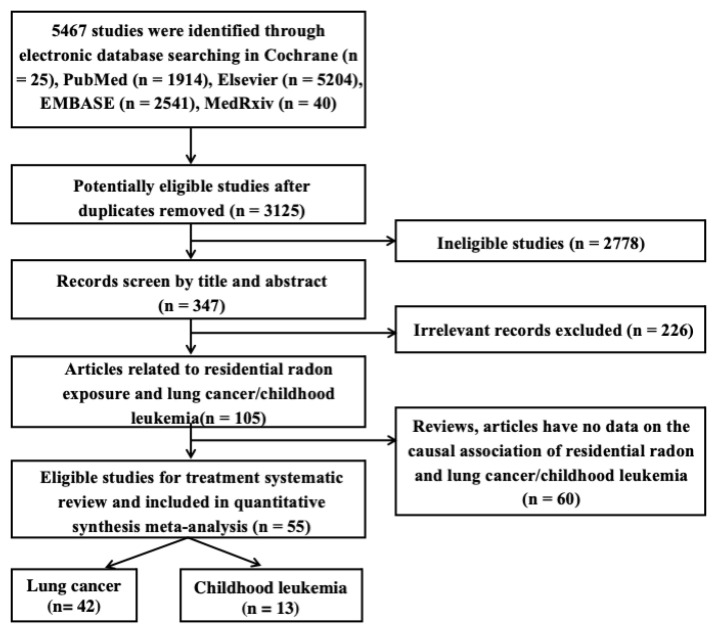
Systematic screening stages of literature review.

Figure 1 shows the flowchart of the process by which the qualified articles were screened. In this study, 9724 different publications were selected by screening through their titles and abstracts based on the searches from the abovementioned databases. After carefully reviewing these articles on the risk of residential radon exposure for human cancers (i.e., lung cancer and childhood leukemia) owing to residential radon exposure, 55 case–control studies were included for a quantitative meta-analysis.

#### Description of Included Studies

The main characteristics of the 55 studies are summarized in Table 1. Among the 55 articles, 42 case–control studies reported the incidence of lung cancer in the participants, and the remaining 13 reported a strong relationship between radon exposure and childhood leukemia. These studies covered the period from 1992 to 2022 and included 48,726 cases and 98,691 control-matched participants. They were conducted in different parts of the world, including Africa (n = 1), Asia (n = 8), North America (n = 15), and Europe (n = 31), thereby ensuring good geo-ethnic representation. In addition, they were conducted over different time periods, including <10 years (n = 39) [21,22,23,24], 10–20 years (n = 10) [25,26,27,28], and 20–40 years (n = 6) [11,29,30,31,32,33], thus providing the high statistical significance for the subgroup analysis based on the period of investigations. To evaluate the adverse effects of residential radon exposure on human health, the researchers compared lung cancer/childhood leukemia rates between the cases and controls based on various categories of radon exposure levels, including at <100 Bq/m^3^ (n = 7), ~100–150 Bq/m^3^ (n = 27), and ≥200 Bq/m^3^ (n = 21). These comparisons were made based not only on radon exposure levels but also the adjustment criteria, especially the sub-criteria of “sex, age, and smoking status” [22,27,34] and “age, socioeconomic, mom’s age, and parental occupational exposure” [12,16,31] for studies of lung cancer and childhood leukemia, respectively. Further, all included studies revealed the primary outcomes by presenting the estimated size effects (i.e., OR/RR) of the incidence of cancers caused by residential radon inhalation. Overall, most studies reported a high association between residential radon exposure and increased lung cancer/childhood leukemia.

### 3.2. Association between Residential Radon Exposure and Human Cancers

#### 3.2.1. Increased Incidence of Lung Cancer

**Figure 2:**
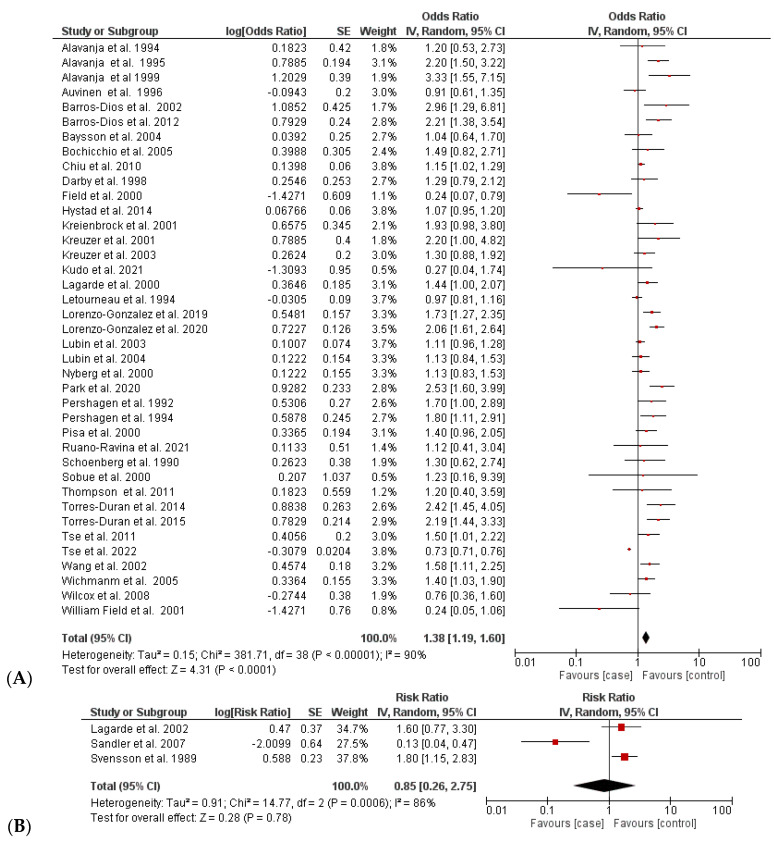
Comparison of incidence of radon-caused lung cancer between case and control groups: (**A**) analysis based on OR values [4,5,13,14,15,21,22,23,24,25,26,29,34,35,36,37,38,39,40,41,42,43,44,45,46,47,48,49,50,52,53,55,56,57,58,59,60,61,62] and (**B**) analysis based on RR values [27,51,54]. (**B**) Red color: OR/RR values of individual studies, Black color: pooled OR/RR of the comparison.

The association between residential radon exposure and increased lung cancer was assessed based on a comparison of the estimated effect sizes (i.e., OR/RR) in 42 case–control studies. The multivariable-adjusted ORs/RRs of lung cancer are shown in Figure 2 and Table 2. A random-effects model was used to calculate the pooled estimated size effects, because substantial heterogeneity was found between these studies (I^2^ = 90%, *p* < 0.00001). The pooled OR of 39 studies showed a statistically significant association between the highest residential radon exposure and increased risk of lung cancer (OR = 1.38 (95% CI 1.19; 1.60)). In contrast, the pooled RR of three case–control studies showed no significant difference in the increase in lung cancer between case and control groups with an overall RR of 0.85 [0.26; 2.73] (I^2^ = 80% and *p* = 0.0006) [14,44,47].

In the sensitivity analysis, removing one study at a time did not significantly affect the pooled OR; it ranged from 1.30 [1.19; 1.62] (I^2^ = 91.43%, *p* < 0.0001) to 1.41 [1.27; 1.58] (I^2^ = 67.30%; *p* < 0.0001). However, the heterogeneity was primarily high owing to the significant variation in the individual study regions, sample size, and other effect factors (e.g., smoking status and residential radon level).

#### 3.2.2. Increased Incidence of Childhood Leukemia

**Figure 3:**
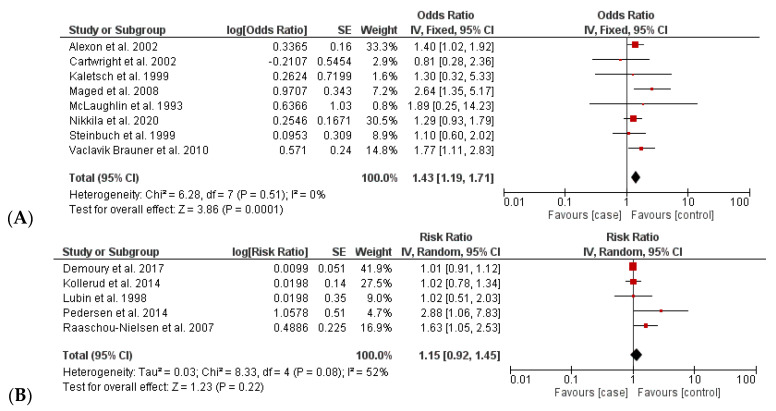
Comparison of incidence of radon-caused childhood leukemia between case and control groups: (**A**) analysis based on OR values [11,12,16,32,63,64,66,67] and (**B**) analysis based on RR values [28,30,31,33,65]. (**B**) Red color: OR/RR values of individual studies, Black color: pooled OR/RR of the comparison.

The pooled estimate of 13 case–control studies for the increase in childhood leukemia induced by residential radon exposure was divided into subcomparisons based on the estimated size effects, namely, OR and RR (Table 2 and Figure 3). The results indicated that the rate of childhood leukemia increased significantly over time with the amount of residential radon exposure with a pooled OR of 1.43 [1.19; 1.72] (I^2^ = 0% and *p* = 0.51). In contrast, the pooled RR suggested no association between radon exposure and cancer (RR 1.15 [0.92; 1.45]; I^2^ = 52%; and *p* = 0.08). The heterogeneities did not exist in the comparison based on OR values, while the comparison based on RR values exhibited a moderate degree of heterogeneity (I^2^ = 0% and 52%).

### 3.3. Subgroup Analysis

In this study, a subgroup analysis was designed to assess the impact of residential radon inhalation on human health based on estimated OR values corresponding to four subcriteria (Table S2). First, the subgroup analysis was stratified by the study population, pooling North America, Europe, Asia, and Africa. Second, the included case–control studies were classified based on the study periods for radon measurement, including <10 years, from 10 to 20 years, and >20 years of radon measurement. Third, classifications were performed based on radon exposure levels of ≤100 Bq/m^3^, 100–150 Bq/m^3^, and ≥200 Bq/m^3^. Finally, the included studies were categorized regarding smoking status of patients, including nonsmokers and smokers.

Regarding the relationship between radon exposure and lung cancer, the subgroup analysis was performed using the random-effects model because the comparisons had considerable heterogeneity (I^2^ > 50%). In the analysis of the study population, a statistically significant increase in lung cancer due to residential radon exposure was observed in the case–control studies conducted in Europe (OR = 1.56 [1.37; 1.78]), but not in those conducted in North America (1.16 [0.95; 1.41], I^2^ = 69.55%, *p* < 0.0001) and Asia (1.24 [0.89; 1.71], I^2^ = 93.78%, *p* < 0.0001) (Table 3). The results showed that studies conducted for ≤10 years or for 10–20 years presented a strong association between lung cancer and radon exposure with pooled ORs of 1.36 [1.16; 1.59] and 1.84 [1.57; 2.15], respectively. An association was also confirmed when comparing case- and control-exposed lung cancer with the high residential radon levels (100–150 Bq/m^3^ and ≥200 Bq/m^3^) (Table 3). Regarding smoking status, residential radon exposure was strongly associated with an increase in lung cancer even among nonsmokers or current smokers with ORs of 1.21 [1.12; 1.31] and 1.38 [1.20; 1.57], respectively. Especially, smokers faced a higher risk of lung cancer than nonsmoker.

The subgroup analyses of studies of childhood leukemia were performed using fixed-effect models because of insignificant heterogeneity (I^2^ < 50%). Statistically significant associations were observed in European studies and studies conducted for <10 years, with pooled ORs of 1.36 [1.12; 1.64] and 1.49 [1.19; 1.86], respectively. Moreover, the adverse effect of a residential radon level of ≥200 Bq/m^3^ on childhood leukemia was confirmed. Notably, all subgroup analyses achieved high statistical significance without heterogeneity (I^2^ = 0%–42.35% and *p* ≥ 0.1).

### 3.4. Dose–Response Analyses

In this study, only 15 included studies provided relevant data regarding lung cancer risk due to a 100 Bq/m^3^ increase in residential radon; therefore, a dose–response analysis was performed based on data extracted from the 15 studies [13,21,29,35,36,39,41,44,45,46,48,59]. Random-effects models were used because of heterogeneity (overall I^2^ = 93.39% and *p* < 0.0001). With a 100 Bq/m^3^ increase in the residential radon level, the risk of lung cancer was not significantly different between the case and control participants (OR = 1.21 [1.01; 1.45], I^2^ = 93%, *p* < 0.00001). Therefore, a dose–response relationship was concluded to exist between all lung cancers and residential radon exposure.

### 3.5. Analysis Bias

**Figure 4:**
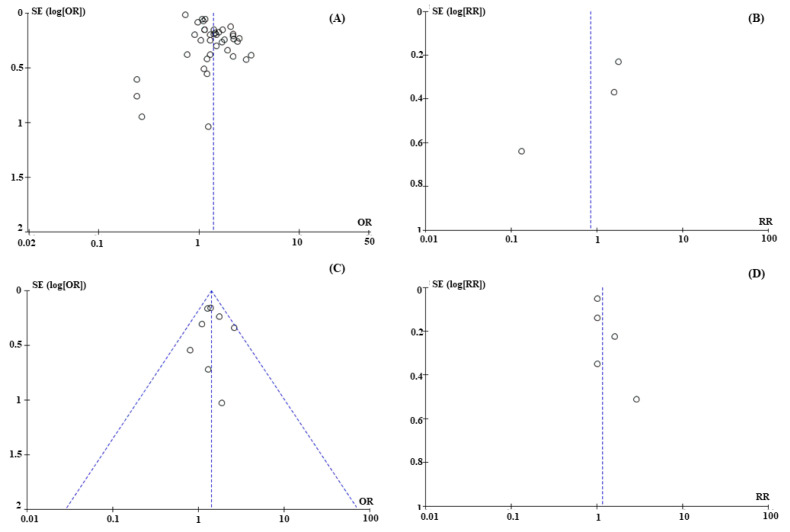
Publication bias of case–control studies related to radon-associated lung cancer and childhood leukemia. (**A**) Lung cancer-based studies with estimated OR values; (**B**) Lung cancer-based studies with estimated RR values; (**C**) Childhood leukemia-based studies with estimated OR values; (**D**) Childhood leukemia-based studies with estimated RR values.

Funnel plots were constructed to detect publication bias. The results indicated that articles with positive OR/RR values have been published, while articles with negative OR/RR values have not been published, leading to an unsymmetrical publication bias (Figure 4A,B). In contrast, although the number of studies of childhood-leukemia-based studies was smaller, the publication was symmetrical and tended to include not only positive outcomes but also several negative ones of each aspect analyzed (Figure 4C,D). Therefore, this meta-analysis should be repeated when large numbers of case–control studies are available in the future to reduce publication bias and analysis heterogeneity.

## 4. Discussion

In this study, the causal associations between residential radon exposure and the increasing incidence of human cancers were assessed through a systematic review and meta-analysis of case–control studies. According to the eligibility criteria, 55 case–control studies with a total of 150,175 participants were enrolled and evaluated, and the results showed that radon exposure was strongly associated with increased lung cancer and childhood leukemia. However, with a 100 Bq/m^3^ increase in the residential radon level, the lung cancer rates were not significantly different between the case and control patients.

For assessing the relationship between radon exposure and lung cancer, 42 case–control studies that provided estimated size effect values (39 ORs and 3 RRs) were selected to perform a meta-analysis. However, the strong relationship was only confirmed through the meta-analysis based on OR values with a pooled OR of 1.36 [1.18; 1.58], even though the analysis had high heterogeneity. The significant heterogeneity in the statistical analysis can be explained by the fact that these included studies were conducted in various regions of the world and under different radon exposure concentrations with different periods of investigation. To reduce the heterogeneity, a subgroup analysis was then designed to reevaluate the relationship based on subcriteria such as the study population, study period, and residential radon exposure. The results revealed a significant difference in lung cancer rate between the cases and controls in Europe (1.56 [1.37; 1.78]) without heterogeneity (I^2^ = 42.28% < 50%), but not in North America and Asia. One out of 10 lung cancer cases in Europe is reportedly attributable to indoor radon exposure, and naturally occurring indoor residential radon may have caused around 19,000 lung cancer deaths in Europe in 2019 [68]. The high incidence of radon-associated lung cancer is relevant to the spatial distribution of indoor radon concentrations across Europe, with high indoor concentrations found in granitic zones and areas with certain rock types [69]. Specifically, energy-efficiency-oriented retrofitting, such as replacing old windows with energy-efficient double-glazed ones, insulating walls, and ceilings or replacing old doors with better sealing ones, may reduce ventilation and increase the building’s airtightness, thus increasing indoor concentrations in a radon-prone area [70]. Owing to the small number of studies on lung cancer and radon conducted in Asia and North America (8 and 11, respectively), these meta-analyses over the two regions had low statistical significance. Second, the subgroup analysis based on the period of investigation also confirmed the causal association of lung cancer and radon exposure, especially for participants exposed within 20 years. In particular, patients exposed to radon for 10–20 years had a higher incidence of lung cancer compared with that of patients exposed for less than 10 years, with pooled ORs of 1.84 [1.57; 2.15] and 1.36 [1.16; 1.59], respectively. Third, a comparison based on the residential radon levels indicated that high radon levels (≥100 Bq/m^3^) could cause significantly adverse effects for the lungs, whereas radon levels ≤100 Bq/m^3^ could be considered less toxic to the lungs. Notably, the highest risk of lung cancer was confirmed when patients were exposed to radon levels of ≥200 Bq/m^3^ followed by radon levels of 100–150 Bq/m^3^ with pooled ORs of 1.37 [1.09; 1.72] and 1.21 [1.13; 1.28], respectively. Finally, the analysis of smoking status indicated a strong association between radon and lung cancer. The results revealed that in the same areas with high radon concentrations, smokers had higher lung cancer incidence than nonsmokers (ORs of 1.38 [1.20; 1.57] > 1.21 [1.12; 1.31], respectively). Thus, it can be said that radon and smoking can be considered to have a synergistic effect on the occurrence and development of lung cancer.

In addition, the impact of residential radon on childhood leukemia was also analyzed based on the estimated ORs and RRs by two discriminant comparisons. However, adverse events were only confirmed by comparing the OR values with pooled ORs of 1.43 [1.19; 1.71] (I^2^ = 0% and *p* = 0.51). In contrast, the pooled RR of 1.15 [0.92; 1.45] (I^2^ = 52% and *p* = 0.08) from five case–control studies suggested no significant difference between childhood leukemia in the case and control groups. The controversial results may be due to the small size of the analysis, with eight studies reporting OR values versus five studies reporting RR values. Thus, adverse effects must be analyzed when larger sample sizes are obtained in the future. Similar to the lung cancer issues, the impact of radon on childhood leukemia was analyzed based on three subcriteria: study population, period of investigation, and radon level. The results also indicated that European children were more affected by residential radon than North American children, with pooled ORs of 1.36 [1.12; 1.64] and 1.89 [0.25; 14.17], respectively. Investigations with a shorter duration (≤10 years) seemed to yield highly accurate results for predicting adverse effects compared with a longer duration (≥20 years). Notably, the impact of radon exposure was confirmed in participants exposed to high radon levels (≥200 Bq/m^3^) with OR of 1.56 [1.02; 2.39] (I^2^ = 42.35% and *p* = 0.188). Therefore, a high residential radon level over a long period is concluded to be positively associated with childhood leukemia; it mutates cells in the immune system and bone marrow, leading to DNA damage and influencing other biologic mechanisms, and consequently inducing childhood leukemia [71].

Therefore, technical solutions must be found to reduce radon levels in existing buildings. For example, the WHO has recommended several technological solutions, including installing a radon sump system, increasing under-floor ventilation, improving ventilation, preventing radon from passing from the basement into living spaces, and sealing floors and walls [72]. A variety of policy solutions have also been proposed, such as providing information on radon levels and health risks; including radon prevention in building codes; establishing radon concentration reference levels; providing education on subsidies for radon reduction measures; including radon as a risk factor in national strategies related to cancer control, tobacco control, indoor air quality, and energy conservation [72].

Recently, many meta-analyses dealing with this topic have been designed to analyze data extracted from several studies, including case–control, ecological studies, cohort-match control, individual studies, and unpublished studies. For instance, Darby et al. (2004) determined the risk of lung cancer associated with radon exposure through a collaborative analysis of 13 case–control studies in nine European countries [4]. The results showed that lung cancer incidence increased by 0.084 [0.003; 0.158] per 100 Bq/m^3^ increase in radon. Further, residential radon, particularly for smokers and recent ex-smokers, was concluded to be responsible for approximately 2% of all cancer deaths in Europe [73]. Li et al. (2019) collected OR values from 28 case–control studies (13,748 lung cancer cases and 23,112 controls) to evaluate the risk of residential radon across histological types of lung cancer. The meta-analysis provides evidence for a positive and statistically significant relationship between radon exposure and all histological types of lung cancer such as small-cell lung carcinoma, adenocarcinoma, and squamous cell carcinoma with pooled ORs of 2.03 [1.52; 2.71], 1.58 [1.31; 1.91], and 1.43 [1.18; 1.74], respectively [2]. Further, another meta-analysis from Lu et al. (2020) included 10 studies (eight case–control and two cohort studies) involving 12,231 cases and 16,202 controls relevant to domestic radon exposure and childhood leukemia. A weak association was found in the case–control studies relative to that in the cohort studies, with an OR of 1.22 [1.01;1.42] and hazard ratio (HR) of 0.97 [0.81; 1.15], respectively [73]. Moon et al. (2021) estimated the possible causal association between residential radon exposure and leukemia using three types of studies, including case–control (n = 9), ecological (n = 8), and cohort studies (n = 15) [17]. Pooling of nine case–control studies increased the pooled OR by 1.03 [1.00; 1.06] for each 100 Bq/m^3^ radon increase for childhood leukemia [17]. All of the most current meta-analyses were performed with only a few case–control studies that were not large enough to accurately estimate the size effect and the significant difference between cases and controls. Moreover, the studies only described meta-analyses with a collaborative approach to collect case–control studies regarding the investigation period and study region. In particular, our study is the first to focus exclusively on the effect of residential radon on two types of human cancers (i.e., lung cancer and childhood leukemia). This study included the most extensive dataset for both emerging cancers (86,688 participants for lung cancer and 63,487 participants for childhood leukemia) and included up-to-date information relevant to the topic, such as study region, smoking status, levels of radon, and two kinds of measurement outcomes (i.e., OR and RR), expecting to provide highly accurate analytical results and improve the quality of evidence for the causal relationship between cancers and residential radon, compared to the published meta-analysis articles. Furthermore, the results from subgroup analyses had negligible heterogeneity, indicating high statistical significance.

## 5. Limitations of Study

The meta-analysis performed had three primary limitations. First, this study included only case–control studies; these may provide an incomplete overview of the causal association between residential radon and human health. Therefore, this topic should be addressed by analyzing all types of studies, such as cohort studies, ecological studies, and randomized clinical trials in the future. Second, the results may be affected by the classification of radon exposure. Given the different radon concentrations in other regions, the radon exposure classifications designed for each study are also different. Third, because it extracted only the OR/RR values of all lung cancers and all childhood leukemias, the study only investigated the overview of radon-induced cancers. Therefore, detailed conclusions about the incidence of each histological type of lung cancer (e.g., adenocarcinoma, small cell carcinoma, and squamous cell carcinoma) and childhood leukemia (e.g., lymphoid leukemia and myeloid leukemia) could not be drawn.

## 6. Conclusions

This meta-analysis of 55 case–control studies, including a total of 49,805 case patients and 100,370 control patients, suggested that residential radon was a risk factor for lung cancer and childhood leukemia when using estimated OR values for the analysis versus RR values. Notably, participants who lived in Europe or an area with high radon levels (≥100 Bq/m^3^) had a higher incidence of cancers compared to those in other regions (North America, Asia, and regions with radon levels <100 Bq/m^3^). Thus, appropriate technical and policy solutions should be recommended to reduce radon exposure to ensure the health of environmental conditions and residents. Further, although the meta-analysis revealed the high impact of residential radon on human health, the results were limited by their high heterogeneity and unsymmetrical publication bias. Thus, this investigation should be repeated when more relevant articles are available in the future to overcome the current limitations.

## Figures and Tables

**Table 1:** Summary of the characteristics of case–control studies.

References	Study Location	Period of Investigation	Ages	Human Cancers	Case/Control	Exposure Comparison (Bq/m^3^)	Types of Size Effects	Adjustment Criteria
Alavanja et al., 1994 [34]	USA	1986–1992	55–80	Lung cancer	538/1183	>100 vs. <29	OR	Age, sex, and smoking status
Alavanja et al., 1995 [23]	USA	1986–1992	55–75	Lung cancer	618/1402	>200 vs. <50	OR	Age and sex
Alavanja et al., 1999 [21]	USA	1993–1994	65–84	Lung cancer	512/3886	>148 vs. <37 Increase of 100 Bq/m^3^	OR	Age, sex, education, and smoking status
Auvinen et al., 1996 [35]	Finland	1986–1992	54–75	Lung cancer	1055/1544	>200 vs. <50 Increase of 100 Bq/m^3^	OR	Cigarette smoking, intensity, duration, and age
Barros-Dios et al., 2002 [22]	Spain	1992–1994	35–70	Lung cancer	163/241	148 vs. <37	OR	Sex, age, lifetime tobacco use, family history, and habitat
Barros-Dios et al., 2012 [36]	Spain	2004–2008	50–70	Lung cancer	349/513	>148 vs. <50 Increase of 100 Bq/m^3^	OR	Age, sex, and tobacco consumption
Baysson et al., 2004 [37]	France	1992–1998	40–75	Lung cancer	486/984	>200 vs. <50	OR	Age, sex, region, smoking status, and occupational exposure
Bochicchio et al., 2005 [13]	Sweden	1993–1996	35–90	Lung cancer	384/404	>200 vs. <50 Increase of 100 Bq/m^3^	OR	Age, smoking, and diet
Chiu et al., 2010 [14]	Hong Kong	2002–2004	50–70	Lung cancer	279/322	>150 vs. <25	OR	Age, employment, and years of education
Darby et al., 1998 [4]	UK	1988–1993	45–73	Lung cancer	982/3185	200 vs. <25	OR	Age, sex, smoking status, country of residence, and social class
Field et al., 2000 [24]	USA	1993–1997	40–84	Lung cancer	413/614	148 vs. <50	OR	Age, smoking, and education
Hystad et al., 2014 [38]	Canada	1994–1997	59–63	Lung cancer	2390/3507	>50	OR	Age, sex, smoke consumption
Kreienbrock et al., 2001 [39]	Germany	1990–1996	40–80	Lung cancer	1449/2297	>140 vs. <50 Increase of 100 Bq/m^3^	OR	Age and sex
Kreuzer et al., 2001 [15]	Germany	1990–1996	50–74	Lung cancer	58/803	Increase of 100 Bq/m^3^	OR	Age, region, occupational carcinogens, and indoor radon
Kreuzer et al., 2003 [40]	Germany	1990–1997	50–75	Lung cancer	1192/1640	>140 vs. <50	OR	Smoking and asbestos exposure
Kudo et al., 2021 [41]	China	2005–2007	39–77	Lung cancer	30/39	>100 vs. < 50 Increase of 100 Bq/m^3^	OR	Age, smoking, and income
Lagarde et al., 2000 [26]	Sweden	1980–1995	35–74	Lung cancer	258/487	140 vs. <50	OR	Age, sex, and smoking status
Lagarde et al., 2002 [27]	Sweden	1985–1995	40–70	Lung cancer	110/231	140 vs. <50	RR	Age, sex, and smoking status
Letourneau et al., 1994 [42]	Canada	1983–1990	25–76	Lung cancer	738/738	375 per month	OR	Age and sex
Lorenzo-Gonzalez et al., 2019 [5]	Spain	2002–2017	57–78	Lung cancer	523/892	≥200 vs. ≤100	OR	Age, sex, and environmental tobacco-smoke exposure
Lorenzo-Gonzalez et al., 2020 [43]	Spain	2004–2019	54–71	Lung cancer	1842/1862	>200 vs. <50	OR	Age, sex, and never smoker
Lubin et al., 2003 [44]	USA	1992–2000	35–70	Lung cancer	4081/5281	Increase of 100 Bq/m^3^	OR	Age and sex
Lubin et al., 2004 [45]	China	1985–1987	40–75	Lung cancer	1053/1997	200 vs. <100 Increase of 100 Bq/m^3^	OR	Age, sex, and smoking status
Nyberg et al., 2000 [29]	Sweden	1950–1990	40–75	Lung cancer	1042/2364	>116 vs. <78 Increase of 100 Bq/m^3^	OR	Tobacco smoking, socioeconomic status, residential radon, and occupational exposures
Park et al., 2020 [46]	Korea	2015–2018	57–72	Lung cancer	519/519	≥100 vs. 25	OR	Age, sex, and indoor hours, and smoking status
Pershagen et al., 1992 [47]	Swedish	1983–1986	35–70	Lung cancer	210/209	>150 vs. <24	OR	Age, sex, and smoking status
Pershagen et al., 1994 [48]	Sweden	1980–1984	35–74	Lung cancer	1360/2847	>400 vs. <50 Increase of 100 Bq/m^3^	OR	Age and sex
Pisa et al., 2000 [49]	Italy	1991–1997	30–70	Lung cancer	138/291	>200 vs. <40	OR	Age and sex
Ruano-Ravina et al., 2021 [50]	Spain	2018–2019	51–68	Lung cancer	189/747	>200 vs. <50	OR	Sex, age, and education
Sandler et al., 2007 [51]	USA	1996–2006	40–79	Lung cancer	1474/1811	<100	RR	Age and sex
Schoenberg et al., 1990 [52]	USA	1980–1989	35–60	Lung cancer	433/402	>148 vs. <37	OR	Age, sex, and smoking status
Sobue et al., 2000 [53]	Japan	1976–1996	40–80	Lung cancer	28/36	100 vs. <25	OR	Age, sex, occupational exposure, and smoking status
Svensson et al., 1989 [54]	Sweden	1980–1986	40–70	Lung cancer	210/209	>200 vs. <50	RR	Smoking, age, and degree of urbanization
Thompson et al., 2011 [55]	USA	1990–2010	40–70	Lung cancer	200/397	≥250 vs. <25	OR	Smoking, residency, job exposure, income, and education
Torres-Duran et al., 2014 [56]	Spain	2011–2013	61–79	Lung cancer	192/329	200 vs. <100	OR	Age and sex
Torres-Duran et al., 2015 [57]	Spain	2000–2012	50–70	Lung cancer	198/275	>200 vs. <50	OR	Age and sex
Tse et al., 2011 [58]	China	2004–2006	35–79	Lung cancer	1208/1069	>200 vs. <50	OR	Age and sex
Tse et al., 2022 [59]	Hong Kong	2004–2006	35–79	Lung cancer	1069/1208	Increase of 100 Bq/m^3^	OR	Age and sex
Wang et al., 2002 [60]	China	1994–1998	30–75	Lung cancer	768/1659	>300 vs. <50 Increase of 100 Bq/m^3^	OR	Age, sex, prefecture, and tobacco use,
Wichmanm et al., 2005 [61]	Germany	1990–1997	35–75	Lung cancer	2963/4232	>140 vs. <50 Increase of 100 Bq/m^3^	OR	Sex, age, and smoking status
Wilcox et al., 2008 [62]	Canada	1989–1992	50–75	Lung cancer	561/740	150 vs. <25 Increase of 100 Bq/m^3^	OR	Sex and age
William Field et al., 2001 [25]	USA	1981–2001	40–84	Lung cancer	413/614	55–150	OR	Age and sex
Axelson et al., 2002 [63]	Sweden	1980–1989	<20	Childhood leukemia	312/1418	100	OR	Age, sex, and county
Demoury et al., 2017 [28]	France	1990–2009	<15	Childhood leukemia	9056/30000	>89	OR	Family income and age
Kaletsch et al., 1999 [64]	Germany	1988–1993	<15	Childhood leukemia	164/209	70	RR	Age and sex
Kollerud et al., 2014 [30]	Norway	1967–2009	<15	Childhood leukemia	712/674	>100 vs. 50	RR	Socioeconomic
Lubin et al., 1998 [65]	USA	1989–1993	<5	Childhood leukemia	505/443	≥148 vs. <37	RR	Age, income, sex, type of residence, parental smoking habits, and parental occupation
Maged et al., 2008 [66]	Egypt	1996–1998	2–14	Childhood leukemia	50/110	>90 vs. <40	OR	Age and sex
McLaughlin et al., 1993 [11]	USA	1950–1988	<14	Childhood leukemia	112/890	>50 vs. 0	OR	Parental occupation exposure
Nikkilä et al., 2020 [32]	Finland	1990–2011	2–7	Childhood leukemia	1093/3279	37	OR	Age, parental occupation
Pedersen et al., 2014 [31]	France	1968–1991	<15	Childhood leukemia	879/1621	≥42	RR	Socioeconomic status, maternal age, birth order
Raaschou-Nielsen et al., 2007 [33]	Denmark	1968–1994	<14	Childhood leukemia	860/1720	<260	RR	Age and sex
Steinbuch et al., 1999 [16]	USA	1989–1993	<15	Childhood leukemia	173/254	>100 vs. <37	OR	Sex, age, maternal education, family income, and maternal race
Cartwright et al., 2002 [67]	UK	1992–1996	<14	Childhood leukemia	2226/3773	>200 vs. <24	OR	Age and sex
Vaclavik Brauner et al., 2010 [12]	Denmark	1986–1994	<20	Childhood leukemia	985/1969	>200 vs. <20	OR	Birth order, mother’s age, and electromagnetic fields

**Table 2:** Summary of pooled estimated size effects (95% CI) of case–control studies.

Human Cancer	Types of Estimate Size Effect	No. of Studies	No. of Cases	No. of Controls	Pooled Estimated Size Effect	I^2^	*p* Value
Lung cancer	OR	39	30,884	51,759	1.38 [1.19; 1.60]	90	<0.00001
	RR	3	1794	2251	0.85 [0.26; 2.74]	86	0.0006
Childhood leukemia	OR	8	12,053	36,804	1.43 [1.19; 1.72]	0.00	0.51
	RR	5	50,127	46,360	1.15 [0.92; 1.45]	52	0.08

**Table 3:** Pooled estimated size effects (95% CI) regarding subgroupanalysis of case–control studies.

Subgroup Analysis	Lung Cancer				Childhood Leukemia			
	No. of Studies	OR (95% CI)	I^2^ (%)	*p* Value	No of Studies	OR (95% CI)	I^2^ (%)	*p* Value
All studies	39	1.38 [1.19; 1.60]	90	<0.00001	8	1.43 [1.19;1.71]	0.00	0.51
*Study population*								
North America	11	1.16 [0.95; 1.41]	69.55	<0.0001	1	1.89 [0.25; 14.17]	–	–
Europe	20	1.56 [1.37; 1.78]	42.28	0.025	6	1.36 [1.12; 1.64]	0.00	0.739
Asia	8	1.24 [0.89; 1.71]	93.78	<0.0001	–	–	–	–
Africa	–	–	–	–	1	2.64 [1.30; 5.18]	–	–
*Period of study (years)*								
<10	32	1.36 [1.16; 1.59]	90.78	<0.0001	6	1.49 [1.19; 1.86]	12.41	0.336
10–20	6	1.84 [1.57; 2.15]	0.00	0.545	–	–	–	–
>20	1	1.13 [0.83; 1.55]	–	–	2	1.30 [0.95; 1.79]	0.00	0.714
*Level of radon exposure (Bq/m^3^)*								
<100	–	–	–	–	4	1.44 [0.97; 1.78]	18.05	0.301
100–150	23	1.21 [1.13; 1.28]	63	<0.0001	2	1.48 [0.65; 3.35]	0.00	0.831
≥200	16	1.37 [1.09; 1.72]	91	<0.00001	2	1.56 [1.02; 2.39]	42.35	0.188
*Smoking status*								
Nonsmoker	8	1.21 [1.12; 1.31]	80	<0.0001	–	–	–	–
Smokers	31	1.38 [1.20; 1.57]	83	<0.00001	–	–	–	–

## Data Availability

Data is unavailable due to privacy.

## Supplementary Materials

The following supporting information can be downloaded at: https://www.mdpi.com/article/10.3390/ijerph20010097/s1, Table S1: Quality assessment of included case–control studies based on the Newcastle–Ottawa Scale (NOS) guideline; Table S2: Characteristics of case–control studies for subgroup analysis.
