# Supplementary material for: Human Health Impacts of Residential Radon Exposure: Updated Systematic Review and Meta-Analysis of Case–Control Studies
Source: Int J Environ Res Public Health. 2022 Dec 21;20(1):97. doi: 10.3390/ijerph20010097 (PMC9819115; doi:10.3390/ijerph20010097)
Supplement: Supplementary file 1 [file ijerph-20-00097-s001.zip › ijerph-2065806-supplementary.pdf]

Supporting Information

Human Health Impacts of Residential Radon Exposure: Up-dated Systematic Review and Meta-analysis of Case-Control Studies

Table S1. Quality assessment of included case-control studies based on the Newcastle-Ottawa Scale (NOS) guideline

Studies	Selection of study				Comparability		Ascertainment of exposure		
	Definition of cases	Representative-ness of cases	Selection of controls	Definition of controls	Treatment outcome	On other risk factor	Ascertainment of exposure	Same method of ascertainment for cases and controls	Non-response rate
**LUNG CANCER**									
Alavanja *et al.* 1994 [34]	★	★	★	★	★			★	
Alavanja *et al.* 1995 [23]	★		★	★			★		★
Alavanja *et al* 1999 [21]	★			★	★		★	★	
Auvinen *et al.* 1996 [35]		★	★		★			★	★
Barros-Dios *et al.* 2002 [22]		★	★	★	★			★	
Barros-Dios *et al.* 2012 [36]		★		★			★	★	
Baysson *et al.* 2004 [37]	★		★		★				★
Bochicchio *et al.* 2005 [13]	★			★		★	★	★	
Chiu *et al.* 2010 [14]		★	★		★		★		
Darby *et al.* 1998 [4]		★		★	★			★	★
Field *et al.* 2000 [24]	★		★	★		★			★
Hystad *et al.* 2014 [38]	★		★		★		★	★	★

Kreienbrock *et al*. 2001 [39]		★		★	★			★	
Kreuzer *et al*. 2001 [15]	★			★			★	★	
Kreuzer *et al*. 2003 [40]		★	★	★		★		★	★
Kudo *et al*. 2021 [41]		★		★			★		★
Lagarde *et al*. 2000 [26]	★		★		★			★	
Lagarde *et al*. 2002 [27]	★			★	★	★			★
Letourneau *et al*. 1994 [42]		★	★			★		★	
Lorenzo-Gonzalez *et al*. 2019 [5]		★	★		★		★		★
Lorenzo-Gonzalez *et al*. 2020 [43]	★			★	★		★		
Lubin *et al*. 2003 [44]	★		★	★		★		★	
Lubin *et al*. 2004 [45]		★		★	★			★	★
Nyberg *et al*. 2000 [29]		★	★			★		★	
Park *et al*. 2020 [46]	★		★		★	★	★		
Pershagen *et al*. 1992 [47]		★		★			★		
Pershagen *et al*. 1994 [48]		★	★	★		★	★	★	
Pisa *et al*. 2000 [49]	★			★	★				★
Ruano-Ravina *et al*. 2021 [50]	★		★		★			★	
Sandler *et al*. 2007 [51]	★	★	★				★		
Schoenberg *et al*. 1990 [52]		★		★	★	★		★	
Sobue *et al*. 2000 [53]	★		★		★		★		★
Svensson *et al*. 1989 [54]		★		★	★			★	
Thompson *et al*. 2011 [55]		★		★			★	★	
Torres-Duran *et al*. 2014 [56]	★		★					★	★
Torres-Duran *et al*. 2015 [57]	★	★		★			★	★	

Tse *et al.* 2011 [58]	★		★		★	★		★	
Tse *et al.* 2022 [59]	★		★				★		★
Wang *et al.* 2002 [60]		★	★	★		★			★
Wichmanm *et al.* 2005 [61]	★	★		★			★		
Wilcox *et al.* 2008 [62]		★	★		★		★	★	
William Field *et al.* 2001 [25]		★		★	★			★	
**CHILDHOOD LEUKEMIA**									
Axelson *et al.* 2002 [63]	★		★	★	★			★	
Demoury *et al.* 2017 [28]	★	★		★	★		★		★
Kaletsch *et al.* 1999 [64]	★		★	★	★			★	
Kollerud *et al.* 2014 [30]		★	★		★	★	★		
Lubin *et al.* 1998 [65]		★		★	★		★	★	
Maged *et al.* 2008 [66]	★			★				★	★
McLaughlin *et al.* 1993 [11]	★		★		★	★	★	★	
Nikkilä *et al.* 2020 [32]	★		★	★			★		
Pedersen *et al.* 2014 [31]	★	★		★		★		★	
Raaschou-Nielsen *et al.* 2007 [33]		★	★		★		★		★
Steinbuch *et al.* 1999 [16]		★		★		★			★
Cartwright *et al.* 2002 [67]	★		★		★	★		★	
Vaclavik Brauner *et al.* 2010 [12]	★		★	★		★		★	★

Table S2. Characteristics of case-control studies for subgroup analysis

Studies	Study region	Period of investigation	Smoking status	Level of residential radon (Bq/m^3)	OR (95% CI)	Increased of 100 Bq/m^3 (OR, 95% CI)
**LUNG CANCER**						
Alavanja *et al.* 1994 [34]	North America	≤ 10 years	Smoker	100-150	1.20 [0.89; 1.70]	
Alavanja *et al.* 1995 [23]	North America	≤ 10 years	Nonsmoker	> 200	2.20 [1.50; 3.20]	
Alavanja *et al* 1999 [21]	North America	≤ 10 years	Smoker	100-150	3.33 [1.50; 7.50]	1.32 [0.90; 2.01]
Auvinen *et al.* 1996 [35]	Europe	≤ 10 years	Smoker	> 200	0.91 [0.61; 1.35]	1.01 [0.94; 1.08]
Barros-Dios *et al.* 2002 [22]	Europe	≤ 10 years	Smoker	100-150	2.96 [1.29; 6.79]	
Barros-Dios *et al.* 2012 [36]	Europe	≤ 10 years	Smoker	100-150	2.21 [1.33; 3.69]	1.87 [1.21; 2.88]
Baysson *et al.* 2004 [37]	Europe	≤ 10 years	Smoker	> 200	1.04 [0.64; 1.67]	
Bochicchio *et al.* 2005 [13]	Europe	≤ 10 years	Smoker	> 200	1.49 [0.82; 2.71]	1.30 [1.03; 1.64]
Chiu *et al.* 2010 [14]	Asia	≤ 10 years	Smoker	100-150	1.15 [1.02; 1.29]	
Darby *et al.* 1998 [4]	Europe	≤ 10 years	Smoker	> 200	1.29 [0.79; 2.12]	
Field *et al*. 2000 [24]	North America	≤ 10 years	Smoker	100-150	0.24 [0.07; 0.79]	
Hystad *et al.* 2014 [38]	North America	≤ 10 years	Smoker	100-150	1.07 [0.94; 1.21]	
Kreienbrock *et al.* 2001 [39]	Europe	≤ 10 years	Nonsmoker	100-150	1.93 [0.99; 3.77]	0.13 [-0.12; 0.46]
Kreuzer *et al*. 2001 [15]	Europe	≤ 10 years	Nonsmoker	100-150	2.22 [1.00; 5.05]	
Kreuzer *et al.* 2003 [40]	Europe	≤ 10 years	Nonsmoker	100-150	1.30 [0.88; 1.93]	
Kudo *et al.* 2021 [41]	Asia	≤ 10 years	Smoker	100-150	0.27 [0.04; 1.74]	0.35 [0.07; 1.74]
Lagarde *et al.* 2000 [26]	Europe	10-20 years	Nonsmoker	100-150	1.44 [1.00; 2.11]	
Letourneau *et al.* 1994 [42]	North America	≤ 10 years	Nonsmoker	> 200	0.97 [0.81; 1.15]	
Lorenzo-Gonzalez *et al.* 2019 [5]	Europe	10-20 years	Nonsmoker	> 200	1.73 [1.27; 2.35]	

Lorenzo-Gonzalez *et al.* 2020 [43]	Europe	10-20 years	Nonsmoker	100-150	2.06 [1.61; 2.64]	
Lubin *et al.* 2003 [44]	North America	≤ 10 years	Smoker	> 200	1.11 [0.96; 1.28]	1.11 [1.02; 1.28]
Lubin *et al.* 2004 [45]	Asia	≤ 10 years	Smoker	100-150	1.13 [0.84; 1.60]	1.12 [0.80; 1.50]
Nyberg *et al.* 2000 [29]	Europe	> 20 years	Smoker	100-150	1.13 [0.83; 1.55]	1.80 [0.85; 1.37]
Park *et al.* 2020 [46]	Asia	≤ 10 years	Smoker	100-150	2.53 [1.60; 3.99]	1.56 [1.03; 2.37]
Pershagen *et al.* 1992 [47]	Europe	≤ 10 years	Smoker	> 200	1.70 [1.00; 2.90]	
Pershagen *et al.* 1994 [48]	Europe	≤ 10 years	Smoker	> 200	1.80 [1.10; 2.90]	1.30 [1.10; 1.61]
Pisa *et al.* 2000 [49]	Europe	≤ 10 years	Smoker	> 200	1.40 [0.96; 2.10]	
Ruano-Ravina *et al.* 2021 [50]	Europe	≤ 10 years	Smoker	100-150	1.12 [0.41; 3.06]	
Schoenberg *et al.* 1990 [52]	North America	≤ 10 years	Smoker	100-150	1.30 [0.62; 2.90]	
Sobue *et al.* 2000 [53]	Asia	10-20 years	Smoker	> 200	1.23 [0.16; 9.39]	0.25 [0.03; 2.33]
Thompson *et al.* 2011 [55]	North America	10-20 years	Smoker	> 200	1.20 [0.40; 3.59]	
Torres-Duran *et al.* 2014 [56]	Europe	≤ 10 years	Smoker	> 200	2.42 [1.45; 4.06]	
Torres-Duran *et al.* 2015 [57]	Europe	10-20 years	Smoker	> 200	2.19 [1.44; 3.33]	
Tse *et al.* 2011 [58]	Asia	≤ 10 years	Smoker	100-150	1.50 [1.00; 2.22]	
Tse *et al.* 2022 [59]	Asia	≤ 10 years	Smoker	> 200	0.74 [0.71; 0.76]	0.74 [0.71; 0.76]
Wang *et al.* 2002 [60]	Asia	≤ 10 years	Smoker	100-150	1.58 [1.10; 2.30]	
Wichmanm *et al.* 2005 [61]	Europe	≤ 10 years	Smoker	100-150	1.40 [1.03; 1.89]	
Wilcox *et al.* 2008 [62]	North America	≤ 10 years	Smoker	100-150	0.76 [0.36; 1.61]	
William Field *et al.* 2001 [25]	North America	10-20 years	Smoker	100-150	0.24 [0.05; 0.92]	
**CHILDHOOD LEUKEMIA**						
Axelson *et al.* 2002 [63]	Europe	≤ 10 years		< 100	1.40 [1.00; 1.90]	
Kaletsch *et al.* 1999 [64]	Europe	≤ 10 years		100-150	1.30 [0.32; 5.33]	
Maged *et al.* 2008 [66]	Africa	≤ 10 years		< 100	2.64 [1.30; 5.00]	

McLaughlin *et al.* 1993 [11]	North America	> 20 years	< 100	1.89 [0.21; 11.80]
Nikkilä *et al.* 2020 [32]	Europe	> 20 years	< 100	1.29 [0.93; 1.77]
Steinbuch *et al.* 1999 [16]	Europe	≤ 10 years	100-150	1.58 [0.58; 4.29]
Cartwright *et al.* 2002 [67]	Europe	≤ 10 years	> 200	0.81 [0.28; 2.36]
Vaclavik Brauner *et al.* 2010 [12]	Europe	≤ 10 years	> 200	1.77 [1.11; 2.82]
